# A *Gly98Val* Mutation in the N-Myc Downstream Regulated Gene 1 (*NDRG1*) in Alaskan Malamutes with Polyneuropathy

**DOI:** 10.1371/journal.pone.0054547

**Published:** 2013-02-05

**Authors:** Camilla S. Bruun, Karin H. Jäderlund, Mette Berendt, Kristine B. Jensen, Eva H. Spodsberg, Hanne Gredal, G. Diane Shelton, James R. Mickelson, Katie M. Minor, Hannes Lohi, Inge Bjerkås, Øyvind Stigen, Arild Espenes, Cecilia Rohdin, Rebecca Edlund, Jennie Ohlsson, Sigitas Cizinauskas, Páll S. Leifsson, Cord Drögemüller, Lars Moe, Susanna Cirera, Merete Fredholm

**Affiliations:** 1 Department of Veterinary Clinical and Animal Sciences, Faculty of Health and Medical Sciences, University of Copenhagen, Frederiksberg, Denmark; 2 Department of Companion Animal Clinical Sciences, Norwegian School of Veterinary Science, Oslo, Norway; 3 Department of Pathology, School of Medicine, University of California San Diego, La Jolla, California, United States of America; 4 College of Veterinary Medicine, University of Minnesota, St Paul, Minnesota, United States of America; 5 Department of Veterinary Biosciences, Research Programs Unit, Molecular Medicine, University of Helsinki and Folkhälsen Research Center, Helsinki, Finland; 6 Department of Basic Sciences and Aquatic Medicine, Norwegian School of Veterinary Science, Oslo, Norway; 7 University Animal Hospital, Swedish University of Agricultural Sciences, Uppsala, Sweden; 8 Department of Veterinary Disease Biology, Faculty of Health and Medical Sciences, University of Copenhagen, Frederiksberg, Denmark; 9 Institute of Genetics, Vetsuisse Faculty, University of Bern, Bern, Switzerland; Instituto de Ciencia de Materiales de Madrid - Instituto de Biomedicina de Valencia, Spain

## Abstract

The first cases of early-onset progressive polyneuropathy appeared in the Alaskan Malamute population in Norway in the late 1970s. Affected dogs were of both sexes and were ambulatory paraparetic, progressing to non-ambulatory tetraparesis. On neurologic examination, affected dogs displayed predominantly laryngeal paresis, decreased postural reactions, decreased spinal reflexes and muscle atrophy. The disease was considered eradicated through breeding programmes but recently new cases have occurred in the Nordic countries and the USA. The N-myc downstream-regulated gene (*NDRG1*) is implicated in neuropathies with comparable symptoms or clinical signs both in humans and in Greyhound dogs. This gene was therefore considered a candidate gene for the polyneuropathy in Alaskan Malamutes. The coding sequence of the *NDRG1* gene derived from one healthy and one affected Alaskan Malamute revealed a non-synonymous G>T mutation in exon 4 in the affected dog that causes a *Gly98Val* amino acid substitution. This substitution was categorized to be “probably damaging” to the protein function by PolyPhen2 (score: 1.000). Subsequently, 102 Alaskan Malamutes from the Nordic countries and the USA known to be either affected (n = 22), obligate carriers (n = 7) or healthy (n = 73) were genotyped for the SNP using TaqMan. All affected dogs had the T/T genotype, the obligate carriers had the G/T genotype and the healthy dogs had the G/G genotype except for 13 who had the G/T genotype. A protein alignment showed that residue 98 is conserved in mammals and also that the entire NDRG1 protein is highly conserved (94.7%) in mammals. We conclude that the G>T substitution is most likely the mutation that causes polyneuropathy in Alaskan Malamutes. Our characterization of a novel candidate causative mutation for polyneuropathy offers a new canine model that can provide further insight into pathobiology and therapy of human polyneuropathy. Furthermore, selection against this mutation can now be used to eliminate the disease in Alaskan Malamutes.

## Introduction

The first cases of inherited polyneuropathy in Alaskan Malamutes were observed in Norway more than 30 years ago. Polyneuropathy in Alaskan Malamutes is one of several canine inherited neuropathies described in 22 breeds of dog that share many features with the human Charcot-Marie-Tooth (CMT) group of diseases [1]. CMT in humans is a heterogeneous group of inherited polyneuropathies characterized clinically by motor weakness and sensory loss. This group of diseases is named after the three clinicians who first described it in 1886 but is also known as hereditary motor and sensory neuropathy (HMSN) [2]. Mutations in more than 40 known genes expressed in Schwann cells and neurons are known to be causative of CMT, and the various forms can have an autosomal recessive, autosomal dominant or X-linked mode of inheritance. The mode of inheritance together with the measured motor and sensory nerve conduction velocity (NCV) provides the basis for classification of the CMT phenotypes into several subgroups [3].

A novel autosomal recessive HMSN was first described in a small gypsy community of Lom in Bulgaria, and designated Hereditary Motor and Sensory Neuropathy-Lom (HMSNL) [4]. This neuropathy has a childhood onset and initial presentation as a gait abnormality with progression to limb weakness, sensory loss, skeletal deformities most notably in the feet, and deafness. Neuropathological changes within peripheral nerve biopsies include severe depletion of myelinated nerve fiber populations, and in younger subjects, hypertrophic changes including onion bulb formations [5]. The HMSNL locus was mapped to a narrow interval on human chromosome 8q24 [4], where the causative mutation was subsequently found to be a nonsense mutation at amino acid residue 148 in the N-myc downstream-regulated gene 1 (*NDRG1)* by Kalaydjieva *et al.* [6]. An inherited polyneuropathy in which the causative mutation is a 10 bp deletion in exon 15 of *NDRG1* has recently been identified in Greyhound dogs. This mutation gives rise to a frameshift and a protein which is longer than the wild type protein [7]. The onset of clinical signs is at three to nine months of age and includes exercise intolerance, progressive ataxia, muscle atrophy and inspiratory stridor.

The first cases of polyneuropathy in the Alaskan Malamute population appeared in Norway in the late 1970’s. Onset of signs was noticed in seven- to 18-months-old dogs. Affected dogs were of both sexes. Presenting clinical signs were exercise intolerance, inspiratory stridor and pelvic limb ataxia. Gait abnormalities progressed to ambulatory paraparesis, in some cases deteriorating to non-ambulatory tetraparesis. Additional examinations revealed decreased postural reactions, decreased to absent spinal reflexes, muscle atrophy and laryngeal paresis. On electromyography (EMG) testing, moderately to severely affected dogs and/or protracted cases had diffuse spontaneous activity, such as fibrillation potentials and positive sharp waves, in proximal and distal muscles in all four limbs. In addition, decreased motor nerve conduction velocities were found. The only aberrant finding on electrophysiology testing in mild and/or early cases was spontaneous activity in EMG of interosseus muscles [8]–[10]. Based on pedigree studies and the result of a test mating, it was suggested that polyneuropathy in Alaskan Malamutes was inherited in an autosomal recessive manner, and the disease was consequently named “hereditary polyneuropathy of Alaskan Malamutes” (AMPN) [9]–[10]. More recently, a number of North American cases expressing many similarities to those seen in Norway were described [11].

AMPN was considered to be virtually eradicated in Scandinavia through breeding programmes as new cases had not been reported for many years. However, a case of AMPN was diagnosed in Denmark in 2009, indicating that the disease had reappeared in Scandinavia. Single cases of AMPN had also occurred in Norway and Sweden during the last decade. The Scandinavian Alaskan Malamute Polyneuropathy survey including research partners from Denmark, Norway and Sweden was therefore initiated in 2010 and has later been extended by collaboration with researchers in Finland and the USA. The main focus for this research was to identify the genetic basis for AMPN.

## Results

### Clinical Characterization of Affected Dogs

Onset of clinical signs was noticed from the age of three to 19 months, (median, 13.5 months). As presenting clinical signs, voice changes and/or noisy breathing predominated - in some cases in combination with paraparesis. A few affected dogs expressed paraparesis as the only presenting clinical sign. Typically, clinical signs slowly progressed to exercise intolerance, gait abnormalities and inspiratory stridor, suggesting the presence of laryngeal paresis or paralysis. Gradually, gait abnormalities progressed from paraparesis and ataxia to tetraparesis and abnormal pelvic limb movements of a “bunny-hopping” nature. Many of the affected dogs had difficulty standing and walking up stairs and eventually collapsed. With progression of the disease, the dogs developed muscle atrophy, primarily in the pelvic limbs, but also in the paraspinal musculature. Electrophysiology testing was in accordance with the results reported by Moe and Bjerkås [9] and Moe [10]. All tested dogs had fibrillation potentials and positive sharp waves in several muscles at EMG testing. The majority of cases tested had reduced motor nerve conduction velocities, with inter-individual variation interpreted as a reflection of the severity and stage of disease.

**Figure 1:**
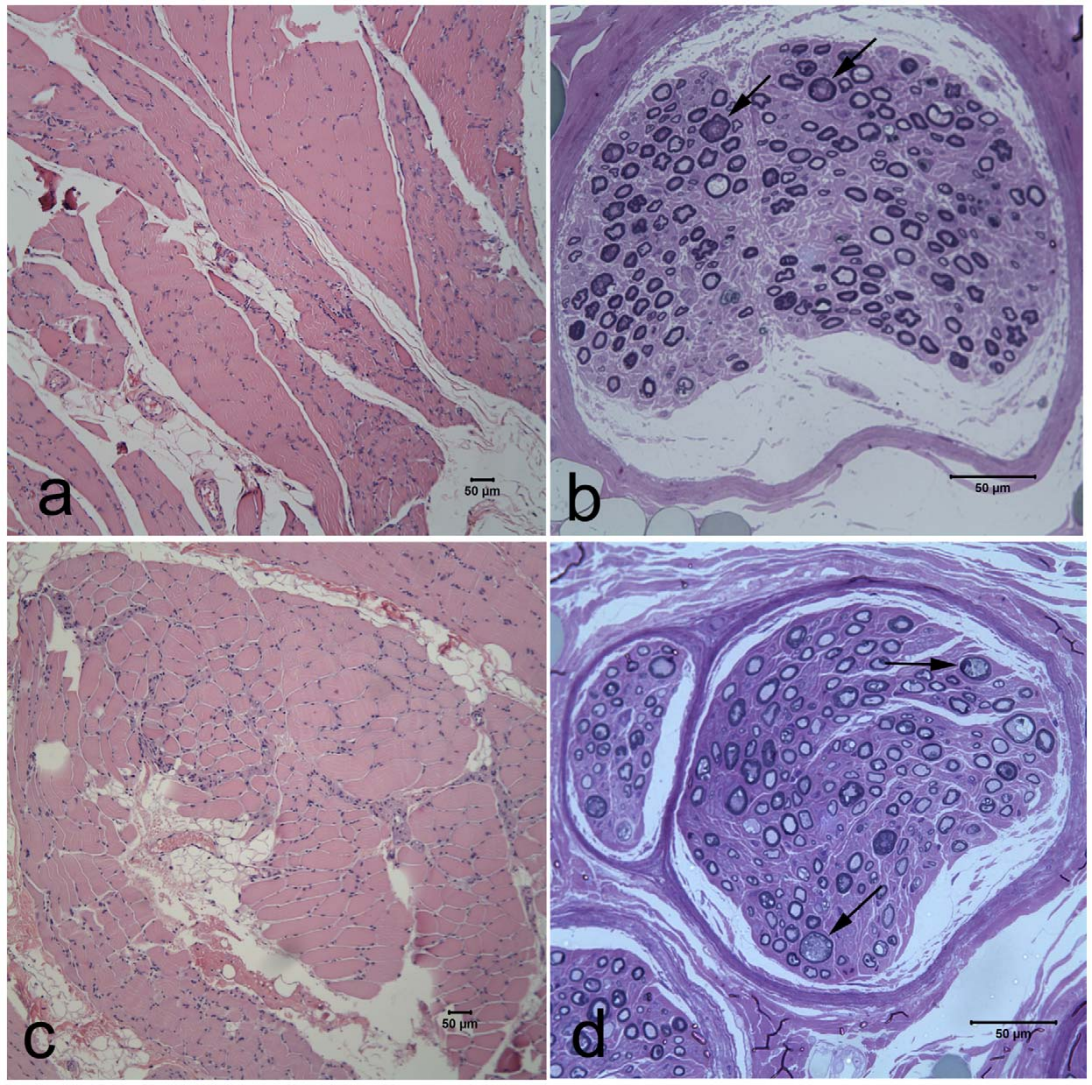
Muscle and peripheral nerve pathology in Alaskan Malamutes with polyneuropathy and a mutation in NDRG1. Representative cranial tibial muscle and peroneal nerve biopsy transverse sections from a two year old female Alaskan Malamute (a,b) and a three year old female Alaskan Malamute (c,d) with histopathological findings consistent with polyneuropathy. Large and small groups of atrophic fibers were present with variable severity and fatty infiltration (a,c. H&E stain). A moderate to marked depletion of myelinated fibers was evident in resin embedded nerve biopsy sections (b,d. Toluidine blue stain). Nerve fiber loss resulted from chronic axonal degeneration (arrows in b,d).

Characteristic histopathological findings in the cranial tibial muscle and fibular nerve are shown from two Alaskan Malamutes (Figure 1). The pattern of muscle fiber atrophy is typical of denervation with angular atrophied fibers occurring in small and large groups and involving both muscle fiber types. Fiber type grouping, indicative of reinnervation, was an inconsistent finding depending on the chronicity of the clinical signs. Variable nerve fiber loss, endoneurial fibrosis and axonal degeneration were consistently found in the fibular nerve and within intramuscular nerve branches.

### Sequencing

**Figure 2:**
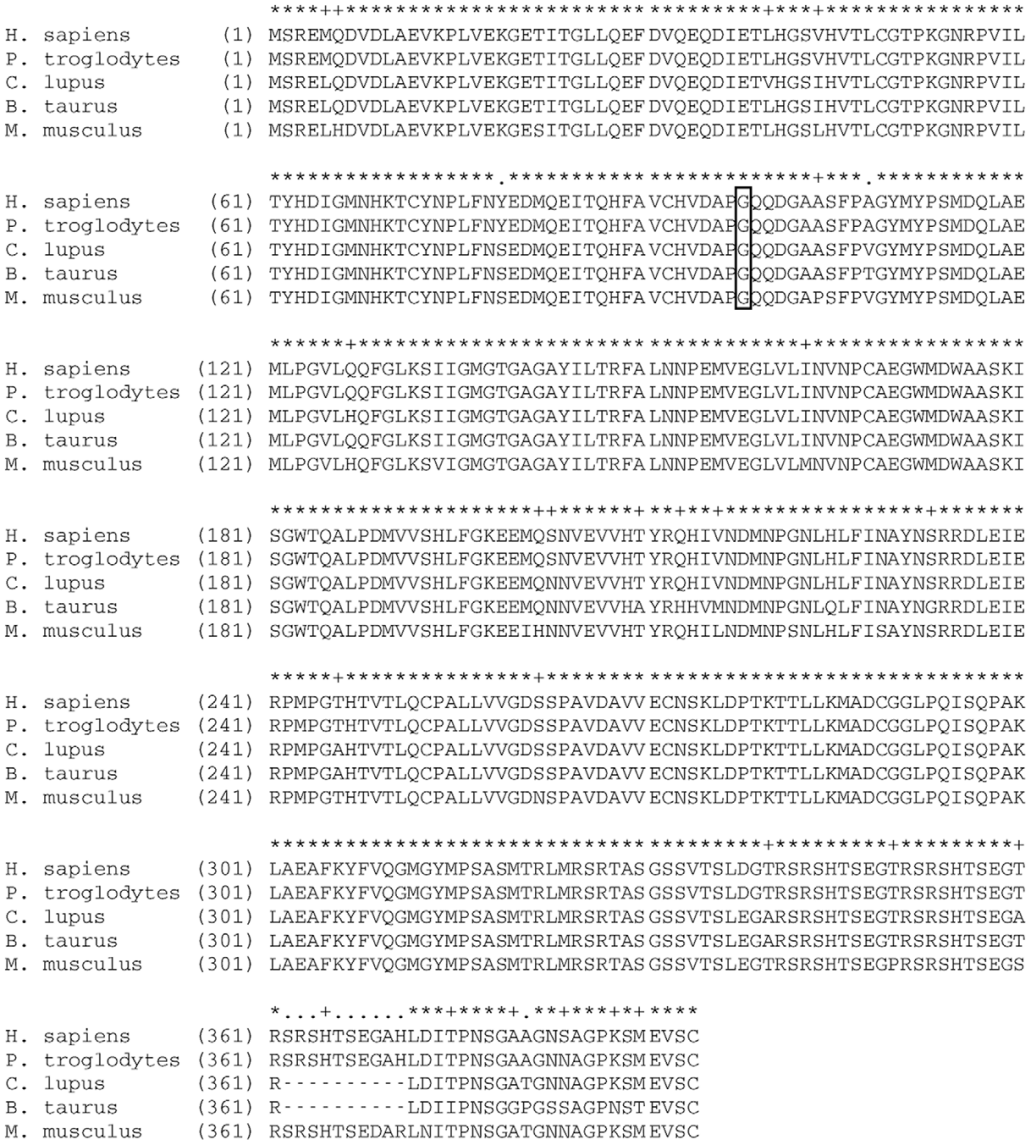
Multiple alignment of mammalian NDRG1 proteins. Alignment of NDRG1 protein sequences from five mammalian species: Homo sapiens, Pan troglodytes, Canis lupus, Bos taurus and Mus musculus shows a sequence similarity of 94.7%. Residue 98 (gly, marked with a frame) which is substituted in Alaskan Malamutes with the G>T mutation, is conserved in the five species.

Since *NDRG1* is mutated in Greyhounds with a similar polyneuropathy, this gene was considered a candidate gene for AMPN. The coding sequence of the canine *NDRG1* is 1152 bp long and comprises 15 exons encoding a protein of 384 amino acids. Except for an inter species variation in the sequence length of exon 15, the coding sequence is well conserved (94.7% sequence similarity) in mammals (see Figure 2 for a multiple protein alignment). The sequencing of the coding regions in one AMPN-affected and one healthy Alaskan Malamute revealed synonymous mutations in exon 1 and 9 and a single non-synonymous G>T (basepair 293) substitution in exon 4. The mutation in exon 4 causes a *Gly98Val* amino acid substitution which was predicted to be probably damaging to the protein function with a score of 1.000 (sensitivity: 0.00; specificity: 1.00) by PolyPhen-2 [12]. To confirm that the mutation resides in a transcribed region of the gene, the canine *NDRG1* transcript was also sequenced revealing the same sequence length and amino acid sequence (JX987297).

### Genotyping

A total of 102 Alaskan Malamute dogs from Denmark, Norway, Sweden, Finland and the USA known to be either healthy (n = 73), obligate carriers (healthy parents of affected offspring) (n = 7) or diagnosed with AMPN (n = 22) were genotyped for the G>T substitution. All affected Alaskan Malamutes had the T/T genotype. The healthy dogs had the G/G genotype except for 13 which had the G/T genotype. All obligate carriers had the G/T genotype. These results indicate complete co-segregation of the mutation with the disease according to an expected autosomal recessive mode of inheritance. Another 201 dogs representing 38 other breeds all had the G/G genotype (see S1 for a list of breeds). Among these, two Leonberger and two Siberian Huskies were diagnosed with polyneuropathy. Four Alaskan Malamutes with neurological signs caused by other diseases (cauda equina syndrome, cervical cord lesion, diabetes and inflammatory polyneuropathy respectively) also had the G/G genotype.

## Discussion

Identification of the genetic basis for AMPN will allow for establishment of an appropriate breeding programme and is therefore beneficial to the general health of the Alaskan Malamute breed. Moreover, this study also highlights the advantages of dogs for comparative genetic studies. Dogs are medically surveyed on a regular basis and the dog population consists of several partially inbred breeds with known, and from time to time new, genetic disorders segregating in most of the breeds. When considering the extensive amount of dog genome resources and rapidly increasing technologies, canine disorders have a great potential for serving as models for analogous human diseases [13].

Various forms of inherited motor and sensory neuropathies have been identified in 22 different dog breeds over the past 50 years [1]. A causative mutation has only been identified in Greyhound dogs with a mutation in the *NDRG1* gene [7].

Our results and observations support the theory that the mutation identified in Alaskan Malamutes causes AMPN: The clinical signs in Greyhound dogs and Alaskan Malamutes with polyneuropathy are comparable. The nonsynonymous G>T substitution causes the substitution (Gly98Val) of a residue that is conserved in mammals and this mutation was predicted to be “probably damaging” by PolyPhen-2. It is therefore reasonable to expect this substitution to have an effect on the function of the protein.Affected dogs were homozygous for the mutation, obligate carriers were heterozygous and healthy dogs were homozygous for the wild type allele (except for 13, which were heterozygous). This is in concordance with autosomal recessive inheritance and also supports the result of the test mating made in Norway in 1983 [10]. Moreover, the fact that *NDRG1* is also mutated in Greyhounds and humans with some forms of polyneuropathy supports the hypothesis that the G>T substitution causes AMPN. If this SNP did not cause disease, it might have existed in other breeds as well. However, it was not detected in any of the 201 dogs representing 38 other breeds. The four Alaskan Malamutes with polyneuropathy with other aetiologies than AMPN illustrate that phenocopies have to be taken into consideration also with this disease. One could speculate that this mutation is not causative but in linkage disequilibrium with another causative mutation in the same region. Therefore protein coding genes within 5MB surrounding the *NDRG1* gene were scrutinized using the annotation in the UCSC browser in the human orthologous region on chromosome 8 (UCSC Human Feb. 2009 (GRCh37/hg19)). In addition to *NDRG1* a total of 13 protein coding genes are annotated in this region. Considering the functional annotation only one gene, *KCNQ3* (potassium channel, voltage-gated, KQT-like subfamily, member 3), is a potential candidate gene. However, since this gene is primarily important for cognitive function and epilepsy [14] we have not studied it further in the context of polyneuropathy in Alaskan Malamutes.

Rentmeister *et al*. [15] recently described AMPN as a polygenic hereditary disease with variable phenotypic expression. However, their diagnostic criteria were very broad and ranged from subclinical forms with signs of marginal degenerative polyneuropathy (based on histopathology) to severe cases supported by EMG and histopathology. Age of onset for dogs in their study varied from seven months to 12 years. In our study the dogs are phenotyped as “affected” only when presenting with the characteristic clinical signs and a defined age of onset (three to 19 months) and most cases were confirmed with histopathology. When using these criteria, we found 100% accordance with the genotyping results and the hypothesis of autosomal recessive inheritance.

A mouse model, *stretcher*, with total *Ndrg1* deficiency (in frame deletion of *Ndrg1* exons 10–14) shows clinical signs characterized by tremor and progressive paralysis of the pelvic limbs from the age of five weeks. Histological examinations reveal demyelination and axonal degeneration of peripheral nerves [16]. Even though the same gene is mutated in the stretcher mouse and the Alaskan Malamutes, the different mutations in two different species are likely to have different effects on NDRG1 function that in turn result in phenotypes that are not completely comparable. Although the *stretcher* and the AMPN phenotypes have different characteristics, the progressive paralysis of the pelvic limbs and axonal degeneration are observed in both species which further supports the association between this phenotype and mutations in *NDRG1*.

*NDRG1* is ubiquitously expressed in various tissues in humans [17] and in rodents and the highest mRNA level is found in the sciatic nerve [18]. Berger *et al.* [18] furthermore show that the *NDRG1* is not expressed in the motor and sensory neurons but highly expressed in Schwann cells where its expression increases shortly after birth and declines in the adult nerve. Axonotomy causes *NDRG1* downregulation indicating an axon-Schwann cell integrity dependent expression [18]. These results show that NDRG1 is important not only for myelination but also for the maturation and maintenance of the neuron. In both the *stretcher* mouse and human cases of HMSNL the demyelination is thought to cause secondary axonal degeneration. In our study of Alaskan Malamutes axonal degeneration without demyelination was observed which also suggests NDRG1 to be central in development and maintenance of the axon.

Inherited polyneuropathy in Greyhound dogs may have an earlier clinical onset (three to nine months) compared to both the AMPN cases of this study (three to 19 months, median: 13.5 months) and in previous studies (seven to 19 months) [7], [10], [11]. Otherwise the clinical and histological findings are similar. One could speculate that the earlier onset in Greyhound dogs is a result of the frameshift mutation having a more severe impact on the protein function than the amino acid substitution observed in Alaskan Malamutes. In both breeds other contributing factors such as the environment, hormonal influences or modifying genes may influence the age of onset and the severity of the disease in individual dogs.

We conclude that the G>T substitution is almost certainly the mutation that causes AMPN.

Since the same mutation is found in affected Alaskan Malamutes from Denmark, Norway, Sweden, Finland and USA, it must have arisen years ago in a common founder. It is therefore reasonable to believe that the same mutation has also caused AMPN in the first Alaskan Malamutes diagnosed by Moe and Bjerkås [9] and by Braund *et al.* [11].

## Materials and Methods

### Ethics Statement

All samples were collected by veterinarians from privately owned dogs with consent from the dog owners and in accordance with the institutional guidelines for animal welfare and ethics. No ethics committee approval was required as the study was conducted in cooperation with veterinarians at small animal clinics and all diagnostic procedures would have been carried out anyway.

### Animal Material

A total of 102 Alaskan Malamute dogs from Denmark, Norway, Sweden, Finland and the USA known to be either healthy (control), obligate carriers or diagnosed with AMPN were included in the study. Control dogs and obligate carriers were checked by veterinarians in the research group. None of those dogs displayed any neurological gait deficits. Affected dogs (n = 22) had a clinical presentation in accordance with AMPN, as described in the results section (clinical characterization). Electrophysiology testing, performed in 16 affected dogs, was consistent with polyneuropathy. In addition, in the majority of the cases the diagnosis was confirmed by histopathology on nerve- and muscle biopsies (n = 13) or post mortem samples of the same tissues (n = 1). Moreover, a panel of 201 DNA samples representing 38 different dog breeds (see S1 for a list of breeds) were included to investigate the genotype in other breeds.

Another four Alaskan Malamutes (two from Norway, two from the USA) were presented to veterinarians in the research group for neurological problems, but were excluded from this study. They were all diagnosed with diseases other than AMPN, explaining their neurological signs (cauda equina syndrome, cervical cord lesion, diabetes and inflammatory polyneuropathy respectively) but were genotyped anyway.

### Muscle and Nerve Biopsies

Biopsies (cranial tibial muscle and fibular nerve) were collected under general anesthesia or in conjunction with euthanasia using an open biopsy procedure. Unfixed muscles were shipped under refrigeration by an express service to a specialized laboratory. Following receipt of tissues, unfixed muscles were flash frozen in isopentane pre-cooled in liquid nitrogen and stored at −80°C until further processed. Following cryosectioning, a standard panel of histochemical stains and reactions including fiber typing was performed on each muscle [19]. Fixed muscle were immersed into 10% neutral buffered formalin, processed routinely and stained with hematoxylin and eosin. Fixed nerve biopsies were resin embedded and evaluated in semi-thin (1 µm) sections.

### Isolation of DNA

EDTA stabilized blood samples were collected from 297 of the dogs. DNA was extracted using a salt precipitation method [20]. In 10 cases for which muscle biopsies were evaluated in cryostat sections, DNA was extracted from frozen archived tissue using the DNEasy Blood & Tissue Kit, Qiagen (Hilden, Germany) following the manufacturer’s recommendations.

### Sequencing of *NDRG1* Exons in Genomic DNA

Each of the 15 exons were PCR amplified one by one in one affected and one healthy dog as described previously [7]. Five µl of each PCR product were run on a 1.5% agarose gel to evaluate the purity and specificity of the PCR reactions. The PCR products were purified using Millipore™ Montage PCR_96_ Cleanup Kit (Billerica, Middlesex County, Massachusetts, USA) according to the manufacturer’s recommendations. Two sequencing reactions, one for each primer, were made for all PCR products using Big Dye® Terminator v3.1 Cycle Sequencing Kit, Applied Biosystems (Foster City, California, USA) and following the manufacturer’s instructions. The sequencing products were purified using Millipore™ Montage SEQ_96_ Sequencing Reaction Cleanup Kit (Billerica, Middlesex County, Massachusetts, USA) according to the manufacturer’s instructions. Sequencing was done using ABI PRISM® 3130 Genetic Analyzer, Applied Biosystems (Foster City, California, USA).

### Sequencing of the Canine *NDRG1* Transcript

After euthanasia of two Belgian Shepard dogs (not affected with polyneuropathy), brain tissue was sampled and snap frozen in liquid nitrogen. RNA was isolated using Qiagen RNeasy Lipid Mini kit (Qiagen, GmbH, Hilden, Germany) following the manufacturer’s recommendations. cDNAs were synthesized from 1 µg of total RNA using ImProm-II™ Reverse Transcription System (Promega, Madison, Wisconsin, USA) and a mixture of random hexamers: oligodT in a ratio of 3∶1, according to the manufacturer’s recommendations.

For amplification of the *NDRG1* transcript two pairs of primers were designed using Primer 3 software [21]: the 5′ UTR primer was designed from a cDNA clone sequence (DN377072.1) (5′UTR L: 5′-TTCGGCAGGTGACAGCAG-3′), the 3′ UTR primer was designed from a cDNA clone sequence (DN401314.1) (3′UTR R: 5′-CGTGAGCCCAGAGTCCAG-3′) and the two internal primers were designed from our previously described genomic exon 7 sequence (Exon 7 L: 5′-AGGGCCTCGTCCTTATCAAC-3′) and exon 9 sequence (Exon 9 R: 5′-GACCACCTCCACGTTGTTCT-3′). PCR reactions with both primer pairs were performed on cDNAs generated from the two brain tissue samples using 2.5 ng/µl cDNA, 2.0 mM MgCl_2_, Qiagen HotStartTaq®DNA Polymerase (Qiagen, GmbH, Hilden, Germany) and an annealing temperature of 60°C. Five µl of each PCR product were run on a 1.5% agarose gel to evaluate the specificity of the PCR reactions. The PCR products were purified using GFX™ PCR- and Gel Band Purification Kit (GE Healthcare Life Sciences, Wauwatosa, Wisconsin, USA).

Two sequencing reactions, one for each primer, were made for all PCR products using Big Dye® Terminator v3.1 Cycle Sequencing Kit, Applied Biosystems (Foster City, California, USA) following the manufacturer’s instructions. The sequencing products were purified using Millipore™ Montage SEQ_96_ Sequencing Reaction Cleanup Kit (Billerica, Middlesex County, Massachusetts, USA) according to the manufacturer’s instructions. Sequencing was performed using ABI PRISM® 3130 Genetic Analyzer, Applied Biosystems (Foster City, California, USA).

### Sequence Analysis

For the sequence assembly, analysis, SNP detection and translation of the cDNA sequence, DNASTAR Lasergene® SeqMan Pro™ (Madison, Wisconsin, USA) was used. PolyPhen-2 [12] was used to evaluate the impact of the amino acid substitution on the protein. Alignment of the human (NP_001128714.1), the chimpanzee (XP_001140704.1), the canine (JX987297), the bovine (NP_001030181.1) and the murine (NP_032707.2) NDRG1 protein sequences was performed using the AliBee Multiple Alignment tool (http://www.genebee.msu.su/services/malign_reduced.html).

### TaqMan

For genotyping of the remaining dogs, a TaqMan assay was designed by Applied Biosystems (Foster City, California, USA) using the following sequence: ACCCAGATGTATTCTTGCCCTACTTGTGCCTCTTCTCTCTCTCCCCTGCCTGTTCTCCAGACAAAACCTGCTACAACCCCCTCTTCAACTCTGAGGACATGCAGGAGATCACACAGCACTTCGCCGTCTGCCATGTGGATGCCCCTG(G/T)CCAGCAGGACGGCGCTGCCTCCTTCCCTGTGGGGTAAGACCCGGAGCCTTGTCCCCAGGAGGGGGACAAGAAAGCCACGCGGGTGGACTGGGGGTGGGGGGTGCGAAGGCAGGCATCACACTGAGT Genotyping was performed according to the manufacturer’s instructions.

## Supporting Information

S1 **Two hundred and one dogs representing these 38 listed dog breeds were genotyped for the G>T mutation.** All had the wild type G/G genotype.
(DOC)
