# Supplementary material for: A Gly98Val Mutation in the N-Myc Downstream Regulated Gene 1 (NDRG1) in Alaskan Malamutes with Polyneuropathy
Source: PLoS One. 2013 Feb 5;8(2):e54547. doi: 10.1371/journal.pone.0054547 (PMC3564917; doi:10.1371/journal.pone.0054547)
Supplement: S1 — Two hundred and one dogs representing these 38 listed dog breeds were genotyped for the G>T mutation. All had the wild type G/G genotype. (DOC) [file pone.0054547.s001.doc]

S1

Dogs representing 38 different breeds genotyped for the G>T mutation:

Australian Sheepdog (2)

Beagle (20)

Bernese mountain dog (2)

Cross bred dogs (4)

Border collie (11)

Boxer (1)

Bull terrier (2)

Bull mastiff (3)

Cairn terrier (9)

Cavalier King Charles Spaniel (2)

Chihuahua (19)

Chinese crested (1)

Chinese crested Powder Puff (1)

Chow-Chow (3)

Cocker Spaniel (6)

Doberman (2)

Miniature Poodle (2)

Miniature Schnauzer (8)

English springer spaniel (2)

Field Trial Spaniel (2)

Finnish Lapphund (5)

Flat coated retriever (7)

Old Danish pointing dog (1)

Golden retriever (8)

Gordon setter (4)

Grand Danois (4)

Groenendael (1)

Hovawart (3)

German Pointing Dog (7)

Irish setter (2)

Irish wolfhound (5)

Icelandic sheep dog (9)

Jack Russell terrier (3)

Kooikerhondje (1)

Labrador retriever (21)

Leonberger (13)

Saint Bernard (2)

Siberian Husky (2)

Rottweiler (1)
